# Exploring climate change vulnerability across sectors and scenarios using indicators of impacts and coping capacity

**DOI:** 10.1007/s10584-014-1162-8

**Published:** 2014-07-12

**Authors:** R. Dunford, P. A. Harrison, J. Jäger, M. D. A. Rounsevell, R. Tinch

**Affiliations:** 1 Environmental Change Institute, Oxford University Centre for the Environment, South Parks Road, Oxford, OX1 3QY, UK; 2 Sustainable Europe Research Institute, Vienna, Austria; 3 School of GeoScience, University of Edinburgh, Drummond Street, Edinburgh, EH8 9XP UK; 4 Iodine SPRL, Brussels, Belgium

## Abstract

Addressing climate change vulnerability requires an understanding of both the level of climate impacts and the capacity of the exposed population to cope. This study developed a methodology for allowing users to explore vulnerability to changes in ecosystem services as a result of climatic and socio-economic changes. It focuses on the vulnerability of Europe across multiple sectors by combining the outputs of a regional integrated assessment (IA) model, the CLIMSAVE IA Platform, with maps of coping capacity based on the five capitals approach. The presented methodology enables stakeholder-derived socio-economic futures to be represented within a quantitative integrated modelling framework in a way that changes spatially and temporally with the socio-economic storyline. Vulnerability was mapped for six key ecosystem services in 40 combined climate and socio-economic scenarios. The analysis shows that, whilst the north and west of Europe are generally better placed to cope with climate impacts than the south and east, coping could be improved in all areas. Furthermore, whilst the lack of coping capacity in dystopian scenarios often leads to greater vulnerability, there are complex interactions between sectors that lead to patterns of vulnerability that vary spatially, with scenario and by sector even within the more utopian futures.

**Electronic supplementary material:**

The online version of this article (doi:10.1007/s10584-014-1162-8) contains supplementary material, which is available to authorized users.

## Introduction

### Context

Assessing the vulnerability of human wellbeing to the impacts of climate change is a key challenge for humanity. The issue is complex and wide-ranging: levels of vulnerability vary across geographical regions, political boundaries and through time, dependent on scenarios of both socio-economic and climatic change (Harley et al. 2008). Vulnerability also varies across sectors and within social groups. For decision-makers and other stakeholders to address these issues, approaches are needed that identify, map and quantify not only the impacts of climate change, but the ability of the exposed societies to cope with them (Vogel and O’Brien 2004).

Many attempts have been made to estimate vulnerability at both global (e.g. Brenkert and Malone 2005) and regional scales (e.g. Emrich and Cutter 2011) by combining measures of climate change impact and societal capacity to address these impacts, mostly in terms of their capability to adapt (adaptive capacity). These vulnerability assessments have taken a number of forms including observations of recent severe events (e.g. Yohe and Tol 2002), expert surveys (Alberini et al. 2006) and indicator-based approaches (Acosta et al. 2013). Furthermore, some studies have focused on the vulnerability of particular sectors to climate and socio-economic change (Villagrán de León 2006), but few explicitly address cross-sectoral interactions . Integrated assessment studies have tackled cross-sectoral impacts of combined climate and socio-economic changes on multiple sectors (Holman et al. 2005), but the ability of society to cope with these impacts is often ignored.

Furthermore, studies which have combined climatic and societal elements have tended to focus on regional-scale adaptive capacity, leading to the critique that such indices poorly reflect the role of individuals within the national/institutional context (Schröter et al. 2004). The concept of *coping capacity*, grounded in a ‘five capitals’ model of resource availability (Porritt 2006), reflects the resources, both tangible and societal, available to help individuals within society cope with the impacts of climate and socio-economic change. However, there have been few attempts to map coping capacity at the European scale, and none that have combined it with cross-sectoral impact modelling for a large number of combined socio-economic and climate scenarios. The aim of this paper is to explore the vulnerability of European human wellbeing by combining indicators of climate change impacts (across multiple sectors and scenarios) with a measure of the available coping capacity (based on indicators of the five capitals). This is done using the integrated assessment platform (IAP) of the CLIMSAVE project.

### Definitions

Vulnerability is an abstract concept and many authors have stressed the need for clear, context-specific definitions of it and related terms (Malone and Engel 2011; Hinkel 2011). Within this paper, “*vulnerability*” is defined as the threat of possible future harm to human wellbeing (Hinkel 2011). It is considered for the European population with respect to the *impacts* of climate and socio-economic change on ecosystem-service provision. The term “*impact*” in this paper is used as shorthand for “negative impacts”; positive impacts (i.e. “benefits”) are not discussed here. The impact may be either “potential impact” (before adaptation) or “residual impact” (following adaptation); “*impact*” is used without adjective where either could apply.

The IAP is used to map these impacts, which are spatially explicit model outputs that reflect the levels of various ecosystem service (ES)-related indicators, such as food provision or the number of people flooded. A society is considered to be more *vulnerable* when the impacts are above (or below) a level that they have the capacity to cope with or adapt to. A terminological distinction is made here between (i) *adaptive capacity* and (ii) *coping capacity. Adaptive capacity* is defined as the amount that a society can reduce climate change impacts (e.g. by technological innovations or behavioural change; Acosta et al. 2013). C*oping capacity* is defined as the resources (both physical and societal) that a society is able to draw on to address the *impact*; coping capacity is related to the levels of the five capitals: human, social, financial, manufactured and natural. Tinch et al. (*this volume*) further discusses the challenges related to defining adaptive and coping capacity and methods for measuring them.

### The CLIMSAVE IAP

The CLIMSAVE project aims to explore both i) the impacts of climate change and socio-economic change and ii) the vulnerability of society to these impacts by using a cross-sectoral modelling approach: the CLIMSAVE integrated assessment platform (IAP). The IAP is an interactive, web-based, cross-sectoral modelling platform that includes interlinked meta-models for a number of sectors including urban development, agriculture, forestry, water provision, flooding and biodiversity (Harrison et al. 2013, 2014). The IAP presents results at a 10’ by 10’ grid cell resolution for the European Union (plus Norway and Switzerland). To represent the range of potential future climates, the IAP contains data for five Global Climate Models (GCMs: CSMK3, MPEH5, HadGEM, GFCM21, IPCM4), four IPCC emissions scenarios (A1b, A2, B1 or B2) and three levels of climate sensitivity (low, medium or high). Pattern scaling is used to combine these data into climate scenarios (Dubrovsky et al. this volume). Any climate scenario can be run for either baseline conditions, the 2020s or 2050s and combined with one of four socio-economic scenarios developed at a series of international stakeholder workshops (involving individuals from government, NGOs, the private sector, research and media, see Kok et al. this volume). These socio-economic scenarios present four futures located at the extremes of two axes of “economic development” and “innovation success” (Gramberger et al. this volume; Kok et al. this volume); they are designed to test the extent to which approaches to coping/adaptation are transferable under divergent socio-economic conditions.

The socio-economic scenarios are as follows:

- *We are the world* (WRW; successful innovation; stable economic growth): characterised by effective government, a focus on well-being and wealth redistribution, reduced inequality and more global cooperation. In 2050 technology has made it possible to live in a CO_2_ neutral society. The redistribution of wealth globally leads to less inequality, more cooperation and a conflict free world.
- *Riders on the storm* (Riders; successful innovation; rollercoaster economic growth): characterised by economic crises countered by investment in green technology, Europe displays a steady green GDP growth and an increase in purchasing power, which is reflected in a population increase. The demand for green technology has also grown with the recovery of the world economy. Although the world economy remains turbulent, Europe is an important player.
- *Icarus* (failed innovation; steady economic decline after 2020): characterised by short-term planning, a stagnating economy, disintegration of social fabric, a shortage of goods and services and a declining population. Eventually some counter-movements start to take root with some signs of a slight economic recovery and post-modern values become more important.
- *Should I stay or should I go?* (SoG; failed innovation; rollercoaster economic decline): characterised by failing to address economic crises, political instability, increased inequality and an insecure, unstable world. Towards 2050 governments start to regulate the use of resources very strictly and instigate power cuts and water rationing. People start exchanging goods, work or services rather than paying for them. Organised crime reaches an all-time high and people live in an insecure and unstable world.

The socio-economic scenarios all have their own input settings, determined at the stakeholder workshops and by IAP experts, for a range of variables which lead to different levels of impact. For example, scenarios where innovation is successful have more optimal values for “water saving due to technological change” and higher “crop yields” as a result of improvements in agronomy. Adaptation is implemented within the IAP by a series of sliders which allow the user to modify the socio-economic scenario variables (for example, increasing the level of flood defence). Adaptive capacity differs across the socio-economic scenarios and this is reflected in the range over which it is possible to change the adaptation variables.

## Method

### Conceptual framework

An ecosystem services framework is used to assess cross-sectoral vulnerability as ecosystem services provide a direct link to human wellbeing (Haines-Young and Potschin 2010). Furthermore, selecting variables across the four main ES categories (provisioning, regulating, supporting and cultural) encourages a focus on indices that are inherently cross-sectoral (such as land use or biodiversity) and indices that reflect non-physical aspects of the environment (such as aesthetics and multi-functionality). In the vulnerability methodology, six ecosystem service indices were selected from the IAP outputs. The selected indices focus on: i) food provision (provisioning); ii) water exploitation (provisioning); iii) flood regulation (regulating); iv) biodiversity (supporting); v) land use intensity (cultural; reflecting the negative consequences on landscape aesthetics associated with landscape intensification); and (vi) landscape diversity (reflecting multi-functionality in terms of the breadth of ecosystem service provision).

### The vulnerability approach

**Fig. 1:**
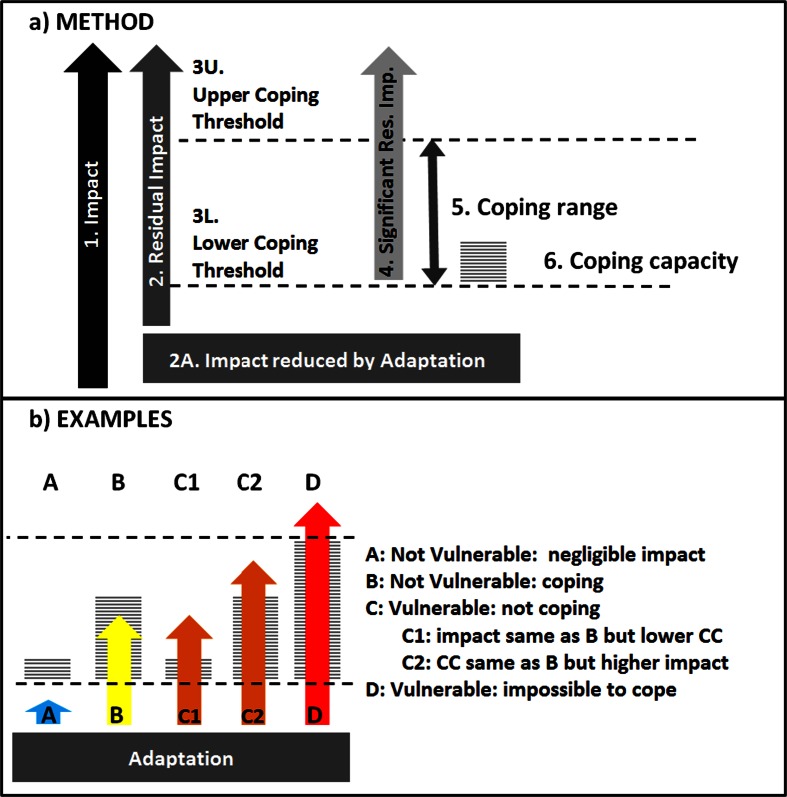
Schematic overview of the vulnerability approach

In the approach reported here, vulnerability comprises three key elements: (i) the potential impact; (ii) the level of adaptation in place to reduce that impact; and (iii) the societal coping capacity available to address the impact that remains after adaptation (Fig. 1a).

**Fig. 2:**
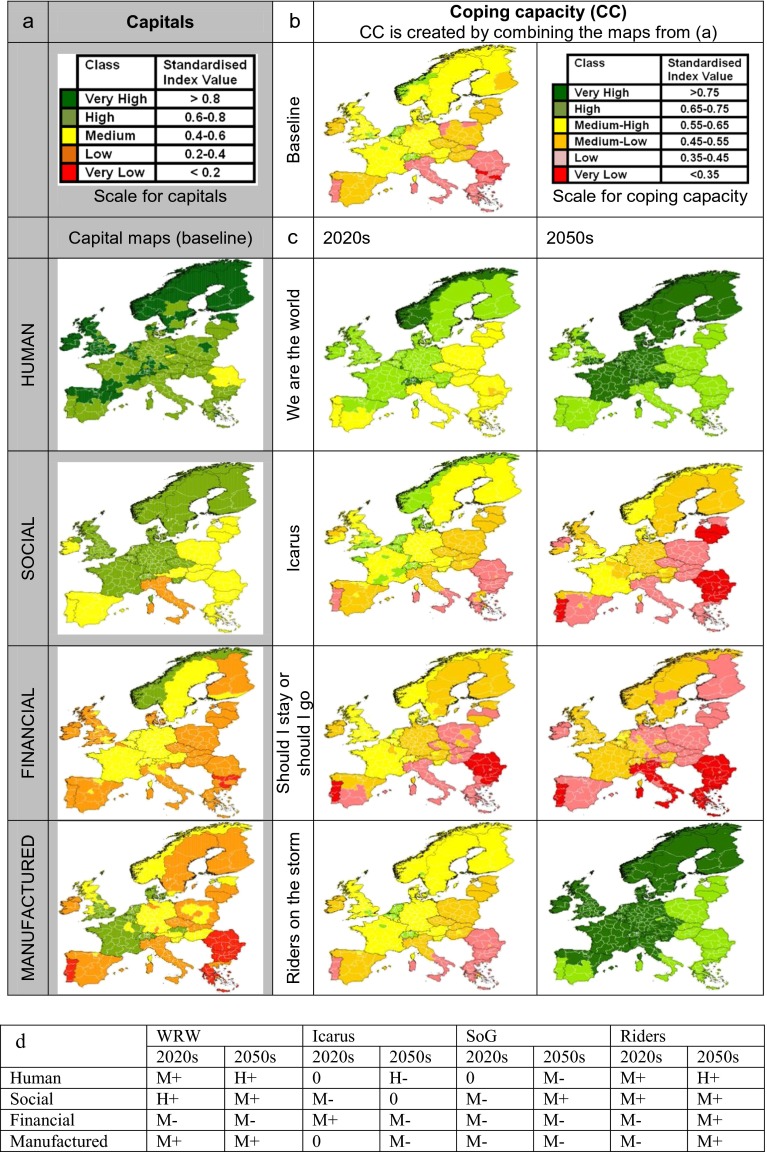
**a** maps of the four capitals for baseline; **b** baseline coping capacity; **c** coping capacity of the four CLIMSAVE socio-economic scenarios (2020s/2050s) **d** the categorisation of capital stocks changes as estimated by stakeholders at the socio-economic scenario workshops. “H” = high and “M” = moderate “+” = positive and “-” = negative

For any combination of socio-economic and climate scenario, the IAP maps for each ES the level of *potential impact* ([1] on Fig. 1a) or the level of *residual impact* [2] following *adaptation* [2A] dependant on whether or not adaptation has taken place. Two thresholds are determined with respect to the level of impact: i) the *lower coping threshold* [3L] below which negligible impacts on wellbeing are expected; and ii) the *upper coping threshold* [3U], above which human wellbeing will be vulnerable irrespective of available capital. Any impact above the lower coping threshold is considered a *significant impact* [4]. Within the ‘*coping range*’ between the two thresholds [5], vulnerability is determined by whether or not the available coping capacity [6] is greater than the significant impact.

A grid-cell is considered to be vulnerable if the impact is either above the upper coping threshold or within the coping range, but greater than the coping capacity. It should be noted that the approach taken summarises vulnerability at a grid cell level, and some sub-regions and members of the population may be vulnerable even in cells identified as coping (and vice versa).

### Quantifying coping capacity

Coping capacity is derived from the levels of five capitals (natural, human, social, financial and manufactured; Porritt 2006). Natural capital (NC) is directly represented by the biophysical modelling. The IAP maps factors such as water, timber and food provision and responds directly to changes in the climate and socio-economic scenarios. As such if more NC is available in a scenario the corresponding sectoral impacts are lower. However, human, social, financial and manufactured capital are not outputs of the biophysical modelling: to ensure their inclusion and avoid the double counting of NC, a coping capacity metric is developed focusing only on these four capitals using an indicator based approach (e.g. Füssel 2010 Acosta et al. 2013). Two variables were selected (from 23 candidates) for each capital following six guiding principles: (i) appropriateness (clear conceptual ties between the variable and the capital); (ii) open access (data within the public domain); (iii) statistical independence (low correlation with other indicator variables); (iv) local scale (relevant to individuals over nations), (v) fixed asset (stocks preferred to flows and rates); and (vi) detailed resolution (finer spatial resolution datasets were preferred).

The eight selected variables, detailed in Online Resource 1, are:

- Human capital: Life expectancy; Tertiary Education.
- Social capital: Income inequality; Help when threatened.
- Financial capital: Household income; Net household savings rate.
- Manufactured capital: Transport and Produced capital.

All datasets were freely accessible, the majority available from Eurostat^1^ with the exception of the World Bank’s ‘produced capital’^2^ and ‘help when threatened’ (Eurobarometer 2005).

An expert panel standardised the indicator variables by defining plausible maxima and minima for each indicator in the 2050s with reference to the stakeholder-developed socio-economic scenarios and current data for Europe and the world. The panel sketched curves to determined breakpoints linking indicator values to a common five-class fuzzy scale from very low (0) to very high (1). Consideration was taken of the form of the relationship as well as the threshold values between the five classes and many of the selected relationships were logistic or inverse logistic reflecting the relative importance of small increases at extremes of the indicator variables (Online Resource 2).

At the scenario-building workshops, stakeholders agreed, for each scenario, the direction and magnitude (‘high’, ‘moderate’ or ‘none’) of change in each of the four capitals for the period 2010–2025 and 2025–2055. A 13-class sliding scale with classes from −6 to +6 was developed to translate these changes into shifts in the indicator variables. For shifts in the first time period ‘moderate’ changes moved one class and ‘high’ changes moved two classes. The second time-period was twice as long as the first and so was given a double-weighting. The extreme values of each class were set with reference to the expert-defined plausible extreme values (again with reference to current data for Europe and the world), and values for each indicator were re-standardised to fit these extremes. For example, a shift of ‘high’ in the 2020s would re-standardise the indicator values between the 2020s maximum and a point at the midpoint of the current EU distribution for that variable (see Online Resource 3).

Following transformation, each pair of indicator variables was averaged to calculate capital variables. This approach has the advantage of simplicity and reflects the expectation that low values in one indicator may be compensated for, to some degree, by high values in another. Adding together shifts between the two time blocks facilitates the representation of cumulative changes – including situations where the direction of change is different. Coping capacity was then calculated as the un-weighted average of the values of the four capitals for each scenario.

### Calculating vulnerability

Vulnerability was calculated for combined climatic and socio-economic scenarios in the absence of adaptation. Vulnerability is therefore calculated for each ES by comparing *significant potential impact* with coping capacity. Four classes of vulnerability were used (Fig. 2b): [A] “not vulnerable, negligible impact”: where potential impact is less than the lower coping threshold; [B] “not vulnerable, coping”: where the significant potential impact is less than the coping capacity; [C] “vulnerable, not coping”: where coping capacity is insufficient to deal with the significant potential impact; and [D] “vulnerable, impossible to cope”: where the potential impact is greater than the upper coping threshold.

Vulnerability was mapped at the grid cell scale for each ES, with the exception of the water exploitation index which is calculated at an amalgamated river basin scale (Wimmer et al. this volume). Two summary statistics were calculated for each ES: (i) the *total vulnerable area* (VA) and (ii) the *number of vulnerable people* (VP). These were calculated at the European scale by summing either (i) the area or (ii) the population of grid cells identified as vulnerable (i.e. in either class [C] or class [D]). Furthermore, cross-sectoral aggregate vulnerability was calculated by counting, for each cell, the number of ES that are classified as vulnerable. The ES are as follows:

- *Food Provision* (VA,VP_FOOD_): based on calories per capita, a grid-cell-based index of self-sufficiency in terms of food provision. This does not consider food imports/exports from outside the grid cell. Note: the IAP’s land use module prioritises food provision, and attempts to ensure European food demand is met.
- *Water Exploitation Index* (WEI; VA,VP_WATER_): water availability for a given river basin following utilisation for human consumption, agriculture and industry.
- *Biodiversity Index* (VA,VP_BIODIVERSITY_): identifies, for a mixed group of 12 representative species, where habitat and climate suitability have changed from baseline.
- *Flood Index* (VA,VP_FLOOD_): based on the number of people affected by a 1-in-100-year flood event; considers both fluvial and coastal flooding.
- *Land-use Intensity Index* (VA,VP_INTENSITY_): represents changes in land use intensity and scores grid cells based on the relative proportions of five land use classes output from the IAP in order of increasing intensity: abandoned land < forestry < extensive agriculture < intensive agriculture < urban. Cultural and aesthetic ES, such as recreation and natural beauty, are assumed to be better correlated with less intensive landscapes.
- *Landscape Diversity Index* (VA,VP_DIVERSITY_): based on the Shannon diversity index applied to six landscape components (arable, intensive agriculture, extensive agriculture, forest, abandoned land and urban). It represents the multi-functionality of the environment; areas with high diversity having access to a greater range of ES. As an index focussed on vulnerability to loss of landscape diversity, vulnerability is highest when a single land use type is present and lowest where there is an even mix of multiple land use types.

Vulnerability indices were calculated for each of the four socio-economic scenarios and run twice using each GCM as: (i) a “high-end climate scenario” (where each GCM is combined with the SRES A1 emissions scenario and a “high” climate sensitivity) and, (ii) a “moderate climate scenario” (where each GCM is combined with the SRES B1 emissions scenario and a “low” climate sensitivity). This resulted in a total of 40 combined climate and socio-economic scenarios. The spatial pattern was mapped for each scenario and the number of vulnerable people (VP) and total vulnerable area (VA) was recorded for the 2050s.

## Results

### Vulnerability

**Fig. 3:**
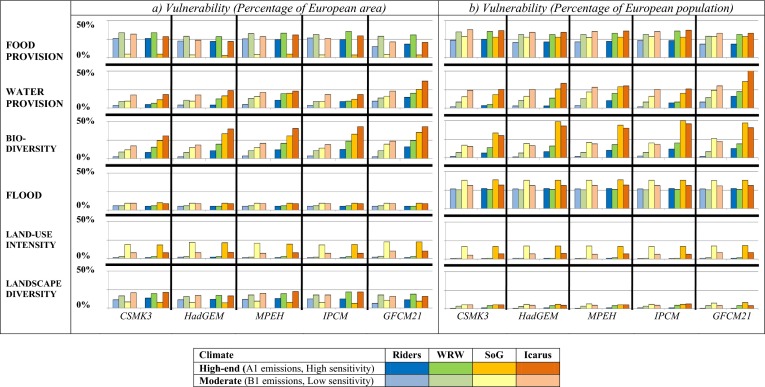
**a** Percentage of European area and **b** population vulnerable. Figures for the six ecosystem service indices by socio-economic and climate scenario

Figure 3 shows the area and number of vulnerable people (i.e. Fig. 1 classes C and D) with respect to each of the ES vulnerability indices for the 40 combined climate and socio-economic scenarios. The relative levels of vulnerability reproduced are consistent with the socio-economic and climate scenarios. In general terms, the dystopian scenarios show greater vulnerability in terms of both the number of vulnerable people and the area vulnerable than the utopian ones for the majority of sectors, particularly in terms of the number of people affected. This reflects the lower significant potential impacts due to the more optimal IAP input settings in the scenarios where innovation is successful, and higher coping capacities where higher capital stocks are available in the utopian scenarios. Similarly in most cases, the more moderate climate scenarios (B1 emissions, low climate sensitivity) have lower vulnerability than the extreme climates (A1 emissions, high climate sensitivity). In addition to these general trends, Fig. 3 shows nuances that reflect the exact combination of climate model, level of climate sensitivity/emissions scenario, socio-economic scenario and sector.

#### Food provision

Irrespective of climate scenario, VA_FOOD_ is considerably lower in the SoG scenario compared to the other socio-economic scenarios (Fig. 3a). In SoG, agricultural yields are low, GDP and irrigation efficiency have decreased, and dietary habits remain focussed on space-intensive meat production. Furthermore, the population grows rapidly (+23 % compared to −9 % in Icarus) so there is much greater demand for food. Consequently, as in SoG there are very few grid cells that do not produce food, grid-cell self-sufficiency is high and VA_FOOD_ is low. In contrast, in WRW, little to no food is produced in Norway and a belt from southern France across the Alps to Hungary and, thus, there are more cells classified as being not self-sufficient in terms of food provision, and thus vulnerable. However, in most climate scenarios, VP_FOOD_ for SoG (Fig. 3b) is comparable with levels of vulnerability in the WRW and Icarus scenarios.

The agricultural model in the IAP does not take food trade between cells into consideration, hence, a grid-cell measure of food provision is used as the best available indicator of food security. As such, this difference between VP and VA in SoG reflects that, although food is being grown wherever possible, urban areas, which do not grow food, remain vulnerable unless they have capital available to access food from areas that do. As an index of vulnerability in terms of the food provisioning ES (rather than food availability) this is reasonable given that urban areas will always be dependent on food producing areas, and those with low levels of available capital will be most vulnerable. Furthermore, with an increasing population in SoG these urban areas become even more dependent on the areas that supply food: and thus more vulnerable in terms of food provision.

There is relatively little difference between the climate scenarios in terms of vulnerability, CSMK3 and MPEH5 show relatively greater vulnerability and HadGEM and GFCM21 show relatively less, but in general the patterns are similar across climate scenarios, and there is little difference between the high and moderate climate scenarios. Again, the differences identified are driven by the extra stress put on the system by climate change: where there is greater stress, such as in the hotter, drier GFCM21 scenario the vulnerability to food provision is projected to be less because food is being grown wherever possible, at the expense of other sectors.

#### Water exploitation

**Fig. 4:**
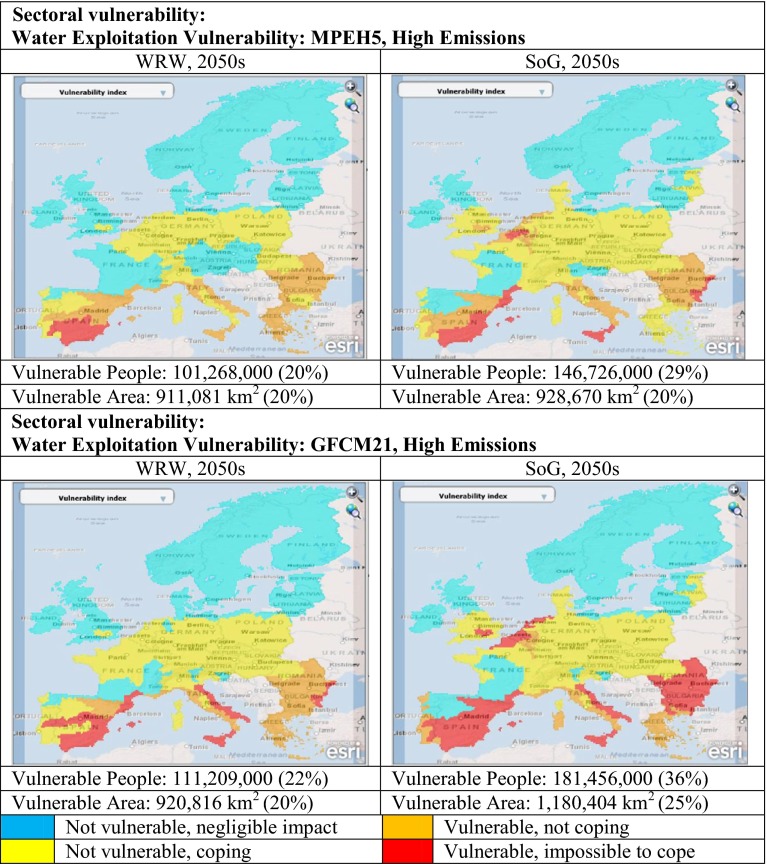
Vulnerability mapping using the Climsave IAP: sectoral vulnerability for selected socio-economic and climate scenarios

In general, the WEI shows increasing vulnerability through the socio-economic scenarios in the order Riders < WRW < SoG < Icarus both in terms of VA and VP. Moderateclimate scenarios show less vulnerability than the high-end scenarios for each GCM. Milder and wetter GCMs (e.g. CSMK3) show less vulnerability than hotter and drier ones (e.g. GFCM21). In some scenarios, such as MPEH5 (and IPCM4) the difference between socio-economic scenarios is much less notable, than in GFCM21 (Fig. 4). Spatial patterns also reflect the complexities of socio-economic interactions. Under MPEH5, WRW does not show vulnerability in the UK, Belgium or the Netherlands that is present in SoG due to improved coping capacity in the former scenario (Fig. 4). However, Greece and the south coast of France are actually *less vulnerable* in the dystopian SoG, than in the utopian WRW. This is due to WRW’s improving GDP (+94 %) leading to lifestyle changes and the use of more water-intensive appliances; a factor most notable in areas with low baseline GDP. Additionally, SoG’s reduced water efficiency leads to irrigation becoming less profitable, compounding the pressures on the agriculture sector and leading to greater areal expansion of agriculture (and concurrent reduction in VA_FOOD_). However, this leaves more water available for other purposes and can reduce water exploitation in some areas. These cross-sectoral interactions help to reveal potential synergies and trade-offs within and between sectors in both the utopian and dystopian scenarios.

#### Biodiversity

Vulnerability to biodiversity loss is hard to conceptualise, particularly in terms of how it would manifest itself in practice. However, it is useful to have an index to consider in relation to the changes in other indicators. Biodiversity vulnerability is absent in all areas where the number of species present at baseline remain the same in the scenario. It increases with the number of species lost due to change in either climate- or habitat-space and when there is not sufficient coping capacity available to address this through active conservation.

VA_BIODIVERSITY_ follows a pattern that reflects decreasing coping capacity across the socio-economic scenarios (Riders < WRW < Icarus < SoG). The same pattern is clear for VP_BIODIVERSITY_ in the two utopian scenarios; however, dystopian SoG has greater VP_BIODIVERSITY_ than Icarus due to SoG’s higher population.

Based on a bioclimatic envelope model, the Biodiversity Index is one of the most climatically sensitive indices: moderate climate scenarios show considerably less VP and VA than their high-end counterparts, irrespective of socio-economic scenario. The level of vulnerability in the CSMK3 high-end climate scenario is about a third less the high-end climate scenarios of the other GCMs (Fig. 3). This is most likely because this scenario leads to the least changes relative to baseline with regard to both climate and land-use, causing fewer negative implications for species.

#### Flooding

In all climate scenarios VA_FLOOD_ and VP_FLOOD_ increase in the order WRW < Riders < Icarus < SoG. Although climatic changes do influence the levels of vulnerability, the differences across climate models are very small (VA/VP_FLOOD_ range < 0.5 %). This results from the small rises in sea-level, the primary driver of coastal flooding, between moderate and high-end climate scenarios (0.12 m and 0.3 m respectively). Fluvial flooding increases in the wetter climate scenarios, but there are few cells which change class between scenarios due to the strong influence of topography, i.e. the flood prone zones. This contrasts with the water and biodiversity indices where there are significant shifts in spatial pattern. Differences between the socio-economic scenarios are more evident reflecting lower coping capacity in the more dystopian scenarios. Changes in population drive the differences in VP_FLOOD_ within the utopian/dystopian scenario pairs; coping capacity drives the differences between the two sets of scenarios.

#### Land-use intensity

SoG has the greatest VA_INTENSITY_ (≈20 %) followed by Icarus (VA_INTENSITY_ ≈ 8 %) with neither of the utopian scenarios showing significant vulnerability (VA_INTENSITY_ < 3 %). Changes in food production (see 3.2.1) drive the majority of this vulnerability. In SoG all available land area is converted to agriculture, at the expense of less intensive land uses and only those areas with little agricultural development (i.e. eastern Sweden) or higher coping capacity (France) are not vulnerable. In Icarus, the declining population reduces the stress on the system and so, despite similarly low levels of coping capacity and failed technological innovations, less area is vulnerable. In the utopian scenarios, there is both a lower need for extreme agricultural intensification and greater coping capacity, which reduces vulnerability significantly. There is very little difference between the climate scenarios in terms of intensity. This is consistent with other studies which show that socio-economic changes are likely to have a greater influence on land use intensity than climate change (Rounsevell et al. 2006).

#### Landscape diversity

As agricultural expansion into new areas is a key driver of the landscape diversity index it shows a similar order of scenarios to food provision and an equally weak response to climate drivers. However, whilst the diversity index is closely linked to the food indicator for the standard settings of the socio-economic scenarios, an analysis of uncertainty associated with the parameter values for the amount of set-aside, agricultural yields and dietary preferences shows that the index is considerably more sensitive to these values than the food index. This is because these parameter values shift the land use distribution between classes within the food-producing land uses (i.e. between arable and the different grassland types) and between food-producing and abandoned land. As such the index has the potential to demonstrate very different responses to the land use intensity and the food provision indices.

### Multi-sectoral aggregate vulnerability

**Fig. 5:**
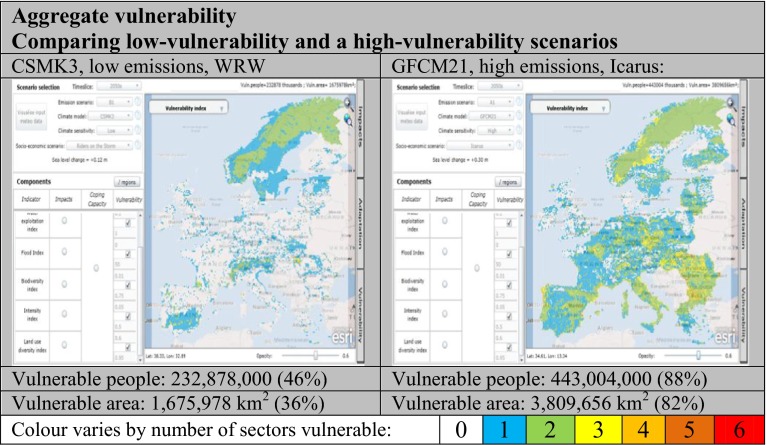
Aggregated cross-sectoral vulnerability mapping using the Climsave IAP

Figure 5 highlights the spatial patterns of cross-sectoral vulnerability for a low and high vulnerability combination of socio-economic and climate scenarios. In the low vulnerability case (moderate climate scenario with CSMK3, WRW, 2050s) there are a few key areas of vulnerability linked mostly to single indicators – for example, southern Spain (water exploitation) and Estonia (food) along with some coastal areas, particularly in northeast Italy (flood). There are very few areas that are vulnerable according to multiple indicators, the most notable being Fennoscandia and the Alps (food and diversity) and pockets of France, Austria and Hungary (food, biodiversity and diversity). This is reflected by the low proportion of Europe vulnerable for at least one indicator both in terms of people (46 %) and area (36 %).

In the high vulnerability case (high-end climate scenario with GFCM21, Icarus, 2050s) European vulnerability is considerably greater with 81 % of the area and 88 % of the baseline population (443,004,000 people) vulnerable in at least one sector. Furthermore, significant areas of Fennoscandia, France, Spain, Italy, Lithuania, Romania, Bulgaria and Greece are vulnerable for more than one indicator. The types of vulnerability differ with geographical area: in Fennoscandia, the vulnerability is due to changes of food and diversity, whilst in southern and eastern Europe, and the areas around Prague and Paris, the vulnerability is to changes of biodiversity and water exploitation. Some areas are vulnerable for three indicators mostly along the coast where they are exposed to floods, but also in areas of Germany, the Czech Republic and Romania where vulnerability to changes of land use intensity is identified.

## Discussion

Indicator-based approaches have been used previously to create maps of adaptive capacity (e.g. Acosta et al. 2013). However, the focus here on coping capacity is novel. Coping capacity represents societal and physical resources available to deal with an emerging crisis. Adaptive capacity takes a much longer-term perspective and associated indicator-based approaches focus on entirely different aspects: (i) the awareness of the population; (ii) the ability of that population to respond; and (iii) the level of action (Greiving et al. 2011; Acosta et al. 2013).

There are similarities between the coping and adaptive capacity approaches: ‘education’, ‘income inequality’, ‘income’ and ‘transport’ are common to both (Online Resource 4); however the reason for their selection is very different. In the adaptive capacity methodologies, education-based indicators such as ‘commitment’, ‘computer skills’ and ‘literacy’ are used as indicators of ‘knowledge and awareness’. In the coping capacity approach, ‘tertiary education’ is seen as a resource – more educated populations are more likely to be better skilled and able to apply those skills to cope with an impact. The differences are not just conceptual: the coping capacity approach does not include top-down indicators such as “R&D expenditure”, “capacity to undertake research”, “GDP per capita”, “government effectiveness” or “democracy”. Instead, the coping capacity indicators focus on the bottom-up, household level, for example, “help when threatened” and “household savings”. They also include a focus on the health of the population which is not usually considered in adaptive capacity approaches.

Nevertheless, the CLIMSAVE coping capacity maps presented here are similar at a general level with the adaptive capacity maps from other studies (Schröter et al. 2004; Greiving et al. 2011; Acosta et al. 2013, see Online Resource 4): Portugal, Spain, southern Italy and Greece are often areas identified as having the least capacity to either adapt or cope and central European and the Nordic countries have comparatively high adaptive/coping capacities. There are also key differences particularly with respect to Fennoscandia, the UK and northern Italy. Using the CLIMSAVE methodology, the Fennoscandian countries and the UK have coping capacity similar to countries in central Europe in most scenarios and time slices. Norway, in particular, has a high ability to cope, driven by high levels of human, social and financial capital, especially by significant levels of household savings. Conversely, both southern and northern Italy have lower coping capacities than in other studies more in line with southern Europe.

A final difference between the methods is that in Schröter et al. (2004)) and Acosta et al. (2013) the measures of adaptive capacity are driven by prescribed relationships identified between the trends in indicator variables, population and GDP. As such, the future projections are driven by changes in population and GDP according to the SRES scenarios and both are projected to grow. The resulting scenarios show very little decrease in adaptive capacity and whilst down-turns are possible, the predominant trend is for a steady increase in adaptive capacity. By contrast, the coping capacity maps show considerably more variety in terms of trend and direction reflecting the diversity of the stakeholder-derived socio-economic futures.

### Vulnerability

Vulnerability can be interpreted differently across sectors. Coping with clear threats to wellbeing (food, water or flooding) is straightforward to conceptualise: areas that cannot cope experience direct impacts on human health. Vulnerability to biodiversity loss, land use intensification or lack of landscape diversity is harder to conceptualise. However, the impacts on mental and spiritual wellbeing and associated knock-on effects on physical health are equally important. Even for the more clear-cut vulnerabilities (e.g. flooding and food) the responses necessary vary greatly. The expert-based threshold approach standardises some of the vulnerability, but the type of coping that would be needed will be different in each case. It is for this reason that the CLIMSAVE IAP is stressed as an exploratory tool to investigate spatial patterns of vulnerability and improve understanding of the driving factors in the context of multiple scenarios and sectors.

The indicator-based methodology presented here is reproducible and comparatively simple. It allows abstract concepts such as the four capitals and coping capacity to be included in quantitative analyses. Furthermore, it directly integrates views from stakeholder discussions in a way that allows complex storylines to be spatially depicted. Additional indicators might capture additional aspects of each capital and reduce the influence of individual variables. However, many of the additional indicator variables considered correlated strongly with those selected and the two-indicator approach maintains the simplicity whilst reproducing patterns that match expectation.

The capitals are combined equally to create coping capacity, yet different ES impacts may require different types of capital to be able to cope. It is conceptually difficult to justify any weighting scheme to determine levels of capital required to cope. A capitals-based analysis of past disaster responses could support quantification, but any weighting will involve subjectivity. Hinkel (2011) argue that the heavy reliance on normative arguments within indicator-based approaches to vulnerability such as this mean that they are only appropriate for local scale studies where the indicator variables can be tied inductively to observed impact datasets. However, the aim here is not to provide definitive maps highlighting vulnerable areas for policy making, the aim is to explore the interactions between sectors within different scenarios, and to provide a tool that allows users to build an understanding regarding the complexity of coupled human-environmental systems (Preston et al. 2011). Many of the decisions involved are necessarily normative, but as the aim is to highlight the interactions between sectors and to better understand the driving factors that lead to different spatial patterns in potential vulnerability the approach is fit for purpose. Further development of the IAP to allow user-defined construction of capitals, coping capacity and vulnerability could further address this by providing the user control over the variables, standardisations and thresholds used, thus providing the user direct control over many of the normative decisions implicit in the current set up. Furthermore, the IAP could also be extended to create a fully dynamic system to enable users to explore the path dependency and spatial evolution of vulnerability.

## Conclusions

This paper presents an assessment of European climate change vulnerability that considers the influence of both climate and socio-economic scenarios across multiple sectors and produces mapped outputs for six key ecosystem services for a number of time slices. Stakeholder-defined storylines are combined with outputs from an integrated bio-physical modelling platform in a manner that is replicable and transferable, and allows the concepts of capitals, coping capacity and stakeholder-derived scenarios to be included within a quantitative system. The findings suggest that, based on current indicators, the north and west of Europe are generally better placed to cope with combined climate and socio-economic impacts than the south and east and that there is scope for improvement in coping capacity in all areas, particularly in terms of stocks of social, financial and manufactured capital. Future vulnerability varies greatly with scenario; however, even in moderate scenarios large proportions of Europe (46 % of the population) are estimated to be vulnerable for at least one ecosystem service. The analysis highlights the importance of cross-sectoral interactions and stresses the significance of socio-economic decisions which can lead to vulnerability irrespective of the climate scenario. By allowing comparative levels of vulnerability to be explored across sectors and scenarios the CLIMSAVE approach provides a flexible tool for decision-makers and other stakeholders to inform their understandings of potential future impacts of climate change and the related vulnerability.

## Electronic supplementary material

Below is the link to the electronic supplementary material.

ESM 1 (DOCX 191 kb)

## Footnotes

1
http://appsso.eurostat.ec.europa.eu

2
http://data.worldbank.org/data-catalogue/wealth-of-nations
