# Supplementary material for: Exploring climate change vulnerability across sectors and scenarios using indicators of impacts and coping capacity
Source: Clim Change. 2014 Jul 12;128(3):339–54. doi: 10.1007/s10584-014-1162-8 (PMC4457356; doi:10.1007/s10584-014-1162-8)
Supplement: Supplementary file 1 — (DOCX 191 kb) [file 10584_2014_1162_MOESM1_ESM.docx]

**Online resource 1:** List of indicator variables used.

**Human Capital**

***Life Expectancy***

The variable reflects European life expectancy at birth. Average life expectancy 2007-2009 has been calculated and is the statistic used for the correlation analyses. The average is based on data for all three years for most locations with the exception of specific NUTS units in Germany, France, Ireland and Italy (see detailed metadata for details).

*Units:* Age (years)

*Scale***:** NUTS2

*Reference***:** Eurostat, 2012, Life expectancy at given exact age (ex). Available online at the address below, accessed: 16/4/2012.

<http://appsso.eurostat.ec.europa.eu/nui/show.do?dataset=demo_r_mlifexp&lang=en>,

***Tertiary Education***

The proportion of “persons aged 25-64 with tertiary education attainment” by sex and NUTS 2 level (%) as a percentage of the total population (M+F) in 2010.

*Scale***:** NUTS2

*Units*: percentage

*Reference***:** Eurostat, 2012, “Population aged 25-64 with tertiary education attainment by sex and NUTS 2 regions”. Available online at the address below, accessed: 16/4/2012.

<http://appsso.eurostat.ec.europa.eu/nui/show.do?dataset=edat_lfse_11&lang=en>

**Social Capital**

***Inequality***

The income quintile share ratio or the S80/S20 ratio is a measure of the inequality of income distribution. It is calculated as the ratio of total income received by the 20 % of the population with the highest income (the top quintile) to that received by the 20 % of the population with the lowest income (the bottom quintile). The values for 2010 were used.

*Scale*: NUTS0

*Units*: no units (ratio)

*Reference*: Eurostat, 2012, S80/S20 income quintile share ratio by gender and selected age group (Source: SILC). Available online at the address below, accessed: 16/4/2012.

<http://appsso.eurostat.ec.europa.eu/nui/show.do?dataset=ilc_di11&lang=en>

***Help when threatened***

The Eurobarometer Social Capital report (2005) has tables dealing with a number of social capital indicators. The proportion of the respondents who said they could call on friends for help when threatened was used in the analysis.

*Scale:* NUTS0

*Units*: percentage

*Reference****:*** Eurobarometer, 2005, Social Capital. Special Eurobarometer N^o^ 223. Available online at the address below, accessed: 16/4/2012.

<http://ec.europa.eu/public_opinion/archives/ebs/ebs_223_en.pdf>

**Financial capital**

***Household Income***

Data were available for disposable income per household following purchasing power standardisation (pps). Data for 2007 were used.

*Scale:* NUTS2

*Units*: Euro pps/ household

*Reference:* Eurostat, 2012, Income of households at NUTS level 2. Available online at the address below, accessed: 16/4/2012.

<http://appsso.eurostat.ec.europa.eu/nui/show.do?dataset=nama_r_ehh2inc&lang=en>

***European net household savings.***

The country’s value for net savings in purchasing power standards per inhabitant were used for the correlation analysis. 2010 data were used.

*Scale:* NUTS0

*Units*: Euro pps/capita

*Reference:* Eurostat, 2012, Income, saving and net lending/ borrowing - Current prices. Available online at the address below, accessed: 16/4/2012.

<http://appsso.eurostat.ec.europa.eu/nui/show.do?dataset=nasa_ki&lang=en>

**Manufactured Capital**

***Infrastructure***

The total length of European road, rail and navigable inland waterways networks for 2009 standardised by NUTS2 area from NUTS 2006 GIS data.

*Scale*: NUTS2

*Units*: km /km^2^

*Reference***:** Eurostat, 2012, Road, rail and navigable inland waterways networks at regional level. Available online at the address below, accessed: 16/4/2012.

<http://appsso.eurostat.ec.europa.eu/nui/show.do?dataset=tran_r_net&lang=en>

***Produced Capital***:

Produced Capital from World Bank data. Produced capital is sum of physical capital and urban land, which is valued at 24 percent of physical capital across all countries. Produced capital is defined as accumulation of investment series (gross capital formation) taking into account depreciation at the rate of 5 percent. 20 years is the service lifetime assumption.

*Scale*: NUTS0

*Units*: $ / capita

*Reference:* World Bank, 2012, Produced Capital. Available online at the address below, accessed: 16/4/2012.

<http://data.worldbank.org/data-catalog/wealth-of-nations>

| **Scale** | **Capital** | **Variable name** | **Form** | **Graph** | **EU current**  Min (*country*)  Max (*country*) | **World current**  Min (*country*)  Max (*country*) | **2020s**  **Europe:Min**  **Max** | **2050s Europe:**  **Min**  **Max** |
| --- | --- | --- | --- | --- | --- | --- | --- | --- |
| N2 | H | Life Expectancy | Logistic | 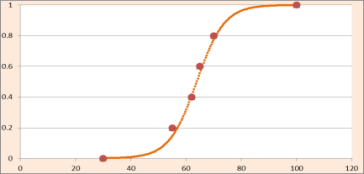 | 72.08 (Lithuania @N0/2)  83.6 (Switzerland @N2) | 31.88 (Swaziland)  89.73 (Monaco) | **60**  **90** | **30**  **100** |
| N2 | H | Tertiary Education | Squared | 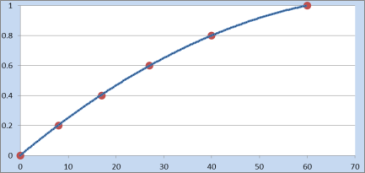 | 13.8 (Malta/ Romania)  53.1 (Finland @N2) | 2 (Sub-Saharan Africa)  42 (Canada) | **10**  **55** | **0**  **60** |
| N0 | S | Income Inequality | Log | 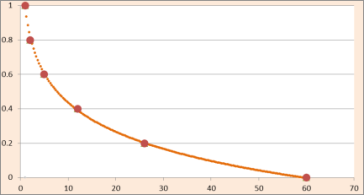 | 3.4 (Slovenia/ Hungary)  7.3 (Lithuania) | 3.4 (Japan)  57.6 (Sierra Leone) | **2**  **10** | **1**  **60** |
| N0 | S | Help When Threatened | Linear | 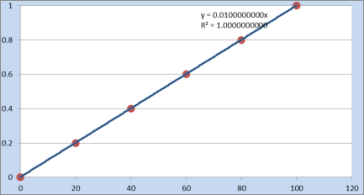 | 15% Hungary  70% (Netherlands/ Sweden) |  | **10**  **75** | **0**  **90%** |
| N2 | F | Household Income | Log | 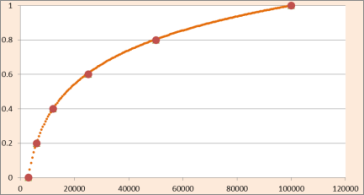 | 3623.8 (Bulgaria @N2)  26324.9 (UK @N2) |  | **€5000**  **€80000** | **€3000**  **€100000** |
| N0 | F | Net household savings rate | Logistic | 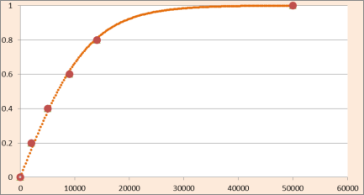 | -2600 (Greece)  9500 (Norway) |  | **€-5000**  **€25000** | **€-5000**  **€40000** |
| N2 | M | Transport | Logistic | 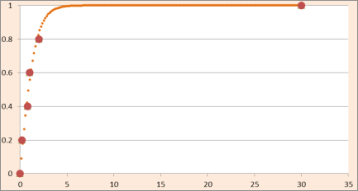 | 4.063 (Belgium @N2)   0.019 (Greece @N2) | Mali ([0.029](http://siteresources.worldbank.org/INTAFRICA/Resources/AICD-Mali_Country_Report.pdf))  Monaco (25.5) | **0.01**  **15** | **0.01**  **30** |
| NO | M | Produced Capital | Logistic | 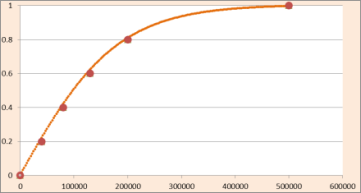 | 6975 (Albania)  213425 (Luxembourg) | 166 (Burundi)  213425 (Luxembourg) | **5040**  **350000** | **0**  **500000** |

**Online resource 2:** Standardising capital indicators: form of relationship between an indicator and capital, average European national statistics and thresholds for the 2020s and 2050s. Scale: N = NUTS. Capital: H = Human; S = Social; F= Financial; M = Manufactured. The x-axis shows real-world values for each indicator whilst the y-axis shows standardised qualitative classes. Points on the graphs show the real-world values that match with thresholds between classes. Numerical values for the qualitative classes are as follows: Very Low (0-0.2); Low (0.2-0.4); Medium (0.4-0.6); High (0.6-0.8) and Very High (0.8-1).

**Online resource 3:** System used to create a flexible coping capacity system.

***Step 1:*** At the scenario-building workshops, stakeholders agreed, for each scenario, the direction and magnitude (‘high’, ‘moderate’ or ‘none’) of change in each of the four capitals for the period 2010-2025 and 2025-2055.

|  | WRW | | Icarus | | SoG | | Riders | |
| --- | --- | --- | --- | --- | --- | --- | --- | --- |
|  | 2020s | 2050s | 2020s | 2050s | 2020s | 2050s | 2020s | 2050s |
| Human | M+ | H+ | 0 | H- | 0 | M- | M+ | H+ |
| Social | H+ | M+ | M- | 0 | M- | M+ | M+ | M+ |
| Financial | M- | M- | M+ | M- | M- | M- | M- | M+ |
| Manufactured | M+ | M+ | 0 | M- | M- | M- | M- | M+ |

***Step 2:*** A 13-class sliding scale with classes from -6 to +6 was developed to translate these changes into shifts in the indicator variables. For shifts in the first time period ‘moderate’ changes moved one class and ‘high’ changes moved two classes. The second time-period was twice as long as the first and so was given a double-weighting.

The shift scores are as follows:

| **Shift** | **Standardisation maximum** | **2020** | **2050** |
| --- | --- | --- | --- |
| H+ | High positive | +2 | +4 |
| M+ | Moderate positive | +1 | +2 |
| 0 | No change | 0 | 0 |
| M- | Moderate negative | -1 | -2 |
| H- | High negative | -2 | -4 |

e.g. a moderate positive score for 2020 (+1) followed by a high negative shift in 2050 (-4) would lead to a total shift score of -3.

***Step 3:*** The extreme values of each class were set with reference to the expert-defined plausible extreme values, and values for each indicator were re-standardised to fit these extremes. For example, a shift of ‘high’ in the 2020s would re-standardise the indicator values between the 2020s maximum and a point at the midpoint of the current EU distribution for that variable.

| **Shift** | **Standardisation maximum** | **Standardisation minimum** |
| --- | --- | --- |
| 6+ | 2050s Max | 2020s Max |
| 5+ | 2050s Max | (Current max + 2020 max)/2 |
| 4+ | 2050s Max | Current Max |
| 3+ | (Current max + 2050 max)/2 | Current Min + 0.75*(Current Range) |
| 2+ | 2020s Max | Current Min + 0.5*(Current Range) |
| 1+ | (Current max + 2020 max)/2 | Current Min + 0.25*(Current Range) |
| 0 | Current max | Current min |
| 1- | Current Min + 0.75*(Current Range) | (Current min + 2020s min)/2 |
| 2- | Current Min + 0.5*(Current Range) | 2020s min |
| 3- | Current Min + 0.25*(Current Range) | (2020s min + 2050s min)/2 |
| 4- | Current min | 2050s min |
| 5- | (Current min + 2020s min)/2 | 2050s min |
| 6- | 2020s min | 2050s min |

***Example:***

The figure below shows an example of this re-standardisation for the life expectancy variable. Current day maximum and minimum for Europe were 81 and 72 years, the expert-defined ‘plausible’ extreme values were 90 and 60 for the 2020s and 100 and 30 for the 2050s. The black bars show the range within which variables are re-standardised for a given value of shift. The solid grey lines delimit the extent of current-day values, the dashed grey lines show the range in the 2020s whereas the full extent of the graph shows the limits in the 2050s. The Black box shows the shifts that are possible in the 2020s, those outside the box can only be achieved in the 2050s. It is not possible to move entirely out of the range of present day values without a shift of at least 4 (either positive or negative), which would require a high increase in either 2020 or 2050 to achieve.


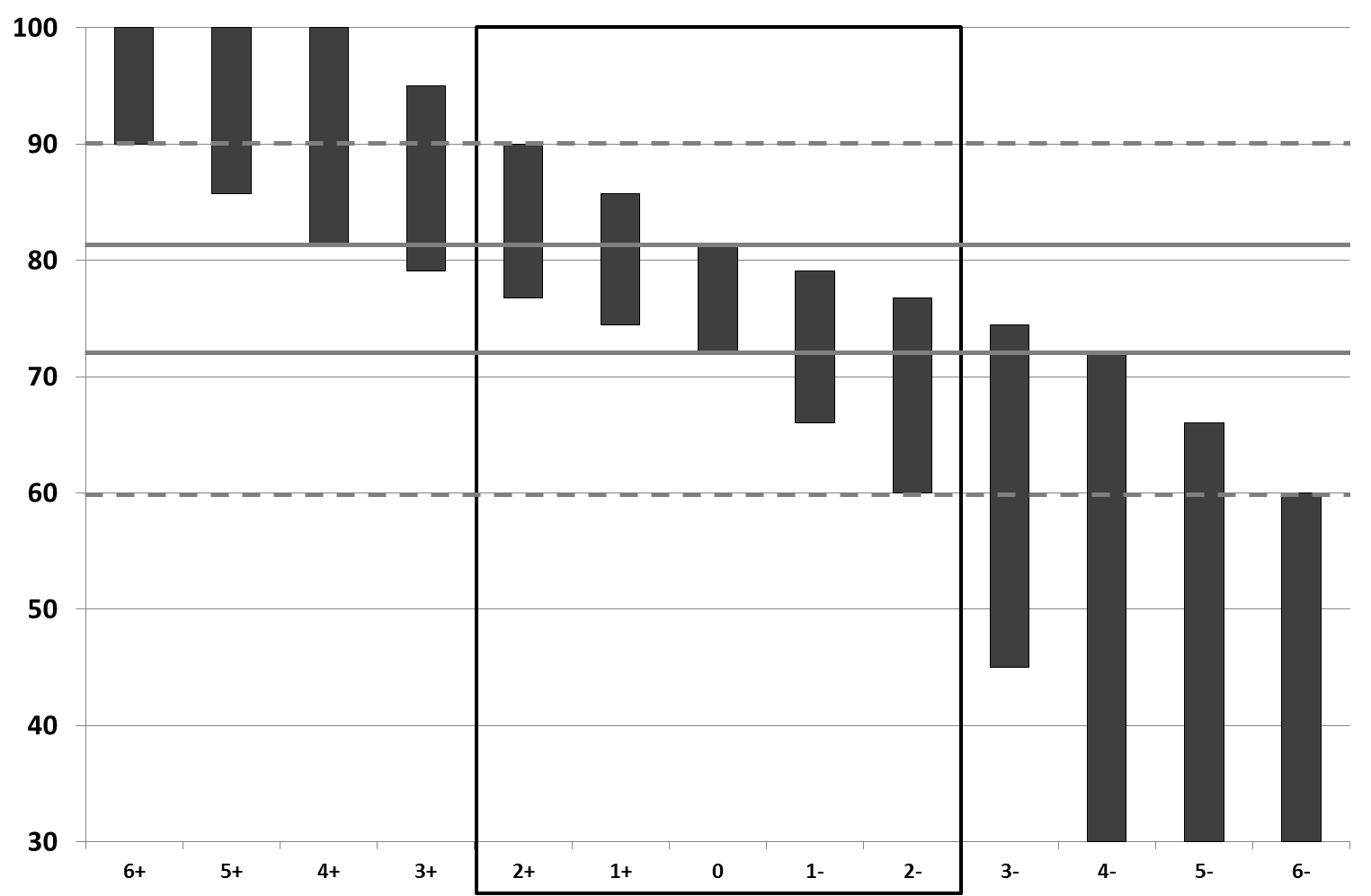


Shift value

Age (years)

| **CLIMSAVE Capital** | **CLIMSAVE Variable** | **Approach** | **Project variable** | **Project Typology** | **Class** |
| --- | --- | --- | --- | --- | --- |
| Human (Health) | Life expectancy | **CLIMSAVE ONLY** | | | Awareness |
| Human (Education) | Tertiary education  (captures similar aspects) | G | Educational commitment | Knowledge and awareness |  |
|  |  | G | Computer skills |  |  |
|  |  | A | Literacy rate |  |  |
|  |  | A | Enrolment ratio |  |  |
| Social (Engagement/  Preparedness) | **Not included in CLIMSAVE** | G | Attitudes towards climate change |  |  |
| Social (Social equality) |  | A | Female activity rate | Equity |  |
| Social (Economic equality) | Income inequality | A | Income inequality |  |  |
| Social (Community networks/ Trust) | Help when threatened  (CLIMSAVE only) | **CLIMSAVE ONLY** | | |  |
| Social (Engagement/  Preparedness) | **Not included in CLIMSAVE** | G | National Adaptation Strategies (NAS) | Institutions | Action |
| Social (Government) | **Not included in CLIMSAVE** | G | Democracy | Institutions |  |
|  |  | G | Government effectiveness | Institutions |  |
| Social (Vulnerability) or  Financial (National) | **Not included in CLIMSAVE** | A/G | Dependency ratio | Flexibility (A)  Economic resources (E) |  |
| Social (Vulnerability) or  Financial (National) | **Not included in CLIMSAVE** | G | Unemployment | Economic resources |  |
| Financial (Household) | Household income | G | Income per capita | Economic resources |  |
| Financial (National) | Correlates highly with household income | A | GDP per capita | Flexibility |  |
|  |  | A | Budget surplus | Economic power |  |
|  |  | A | World trade share | Economic power |  |
| Financial (Household, reserves) | Household savings | **CLIMSAVE ONLY** | | |  |
| Manufactured (Research/innovation) | **Not included in CLIMSAVE** | A | R&D expenditure | Technology | Ability |
|  |  | G | Capacity to undertake research |  |  |
|  |  | G | Resources for technology |  |  |
|  |  | A/G | Number of patents |  |  |
| Manufactured (Transport infrastructure) | Roads, rail and inland waterways | G | Road network density | Infrastructure |  |
| Manufactured (Other infrastructure) | **Not included in CLIMSAVE** | A | Number of doctors |  |  |
|  |  | G | Hospital beds |  |  |
|  |  | G | Sustainable water infrastructure |  |  |
| Manufactured (Assets) | Produced capital | **CLIMSAVE ONLY** | | |  |

**Online Resource 4:** Differences between the CLIMSAVE approach and that of Acosta et al. 2013 (A) and Grieving et al 2011. (G). Coloured entries are those used within CLIMSAVE.
